# Optogenetic Activation of the Excitatory Neurons Expressing CaMKII**α** in the Ventral Tegmental Area Upregulates the Locomotor Activity of Free Behaving Rats

**DOI:** 10.1155/2014/687469

**Published:** 2014-03-10

**Authors:** Songchao Guo, Sicong Chen, Qiaosheng Zhang, Yueming Wang, Kedi Xu, Xiaoxiang Zheng

**Affiliations:** ^1^Qiushi Academy for Advanced Studies, Zhejiang University, Hangzhou 310027, China; ^2^Institute of Biomedical Engineering, Zhejiang University, Hangzhou 310027, China

## Abstract

The ventral tegmental area (VTA) plays an important role in motivation and motor activity of mammals. Previous studies have reported that electrical stimulations of the VTA's neuronal projections were able to upregulate the locomotor activity of behaving rats. However, which types of neurons in the VTA that take part in the activation remain elusive. In this paper we employed optogenetic technique to selectively activate the excitatory neurons expressing CaMKII**α** in the VTA region and induced a higher locomotor activity for free behaving rats. Further behavioral studies indicated that reward learning mediated in the enhancement of the rat locomotor activity. Finally the immunohistochemistry studies explored that the excitatory neurons under the optogenetic activation in VTA were partly dopaminergic that may participate as a vital role in the optogenetic activation of the locomotor activity. In total, our study provided an optogenetic approach to selectively upregulate the locomotor activity of free behaving rats, thus facilitating both neuroscience researches and neural engineering such as animal robotics in the future.

## 1. Introduction

Optogenetics comprises a set of techniques that integrate the opsin genes into specific types of neurons to selectively probe the neural circuits [1, 2] and has currently been introduced into a growing number of neuroscience researches [3–6] and neural engineering systems such as the brain-machine interfaces [7, 8]. The optogenetic technique enables either excitation or inhibition of selected neural populations under the delivery of light at specific wavelengths [9, 10]. Basically the opsin genes are able to express light-sensitive membrane ion channels, produce ion flows, and thus induce or suppress the action potentials in living neural populations [9]. The channelrhodopsin-2 (ChR2), one of the opsin cation channels [10, 11], is typically transduced into excitatory neurons [12, 13] under the guidance of certain promoters such as the calcium-modulin dependent kinase II type-*α* (CaMKII*α*) promoters. The specific neurons with ChR2 and CaMKII*α* expressions would produce action potentials upon the delivery of blue light at a central wavelength of 473 nm [1, 2].

In animal brains, the vast majority of the excitatory neurons expressing CaMKII*α* are distributed in cortex areas and hippocampus [1, 14]. For rats, the CaMKII*α* was also found in deep brain regions, for example, the ventral tegmental area (VTA) within the ventral striatum [15, 16]. The VTA comprises a variety of neurons located on the floor of the midbrain that relate to the mesolimbic dopaminergic system and is widely implicated in the natural reward circuitry of the brain as well as drug addiction and motor activity. Particularly, electrical stimulations of the medial forebrain bundle (MFB), one of the neuronal projections from the VTA region, were able to upregulate the rat locomotor activity as a part of the rat-robot systems [17, 18]. However, the precise mechanisms underlying such rat-robot systems remain unclear, largely due to the extensive and unselective effect of electrical stimulation on all types of neurons. Actually, besides dopaminergic neurons, the VTA also contains glutamatergic neurons [19] that are regarded as a type of excitatory neurons expressing CaMKII*α*. Yet few researches have been engaged in how such excitatory neurons take part in the VTA neuronal activities and whether these neurons alone could influence behavioral conditionings such as locomotor activities.

In this paper we selectively activated the excitatory neurons in the VTA region by optogenetic transductions of the CaMKII*α*-ChR2-mCherry virus into the rat brain. Both histological and electrophysiological methods verified the robustness of the optogenetic manipulations. We found that the optical activations on these VTA neurons were able to induce an enhanced locomotor activity of free roaming rats. Further results revealed that these VTA neurons under optogenetic activation may involve the reward learning mechanisms and were partly dopaminergic, consistent with previous reports on both structural and functional properties of the VTA [20–24]. Taken together, our study based on the optogenetic techniques has provided a novel method to selectively upregulate the locomotor activity of free behaving rats, which could be integrated into the rat-robot systems for precise controls. Also we have further approached the explanation of mechanisms underlying the rat-robot control strategies, thus facilitating future work on both animal robotics and neuroscience researches.

## 2. Material and Methods

### 2.1. Animal Subjects

Adult male Sprague-Dawley rats with 180 ± 10 g body weight were chosen from Zhejiang Academy of Medical Sciences (Hangzhou, China). All the rats were housed in a temperature-controlled room (23 ± 3°C) with access to water and mildly food-deprived to 85% of free-feeding body weight. The rats were kept in individual cages on a 12 h light/dark cycle (lights on at 6 am).

### 2.2. Surgeries

#### 2.2.1. Optrode Implantation

The optrode array device used in this paper was designed in our previous study [25]. The implantation of the array device was implemented by craniotomy on the rats over four postnatal weeks. In general the rats were anesthetized with 1.0% sodium pentobarbital, sheared over the head, and fixed in a stereotaxic apparatus (Stoelting Co., Ltd., USA). For each subject, the craniotomy was centered on the brain region dorsal to the left VTA region: −4.8 mm posterior to the bregma (AP), 1.0 mm lateral to the midline (ML), and −8.0 mm ventral to the cortical surface (DV) according to the atlas of Paxinos and Watson. A series of skull holes were drilled for placement of four skull screws to provide mechanical support as well as common ground references for the implanted array device. In particular, a round craniotomy window with 1.0 mm diameter was drilled for implanting the array device. The dura matter was carefully peeled away using a sterilized needle with operations under microscopy. Then the array device, yet without the optical fiber, was stereotaxically implanted through the craniotomy window into the targeted brain area exactly above the left VTA region. In addition, a ground reference electrode from the array device was bundled firmly on all the skull screws. Finally the craniotomy window was filled with clinical ionized gel for protecting the inner brain and the entire scalp area was covered up using dental acrylic. The rats were allowed to recover for 5–7 days before use in the viral delivery and the optical fiber implantation.

#### 2.2.2. Viral Delivery and Fiber Implantation

**Figure 1:**
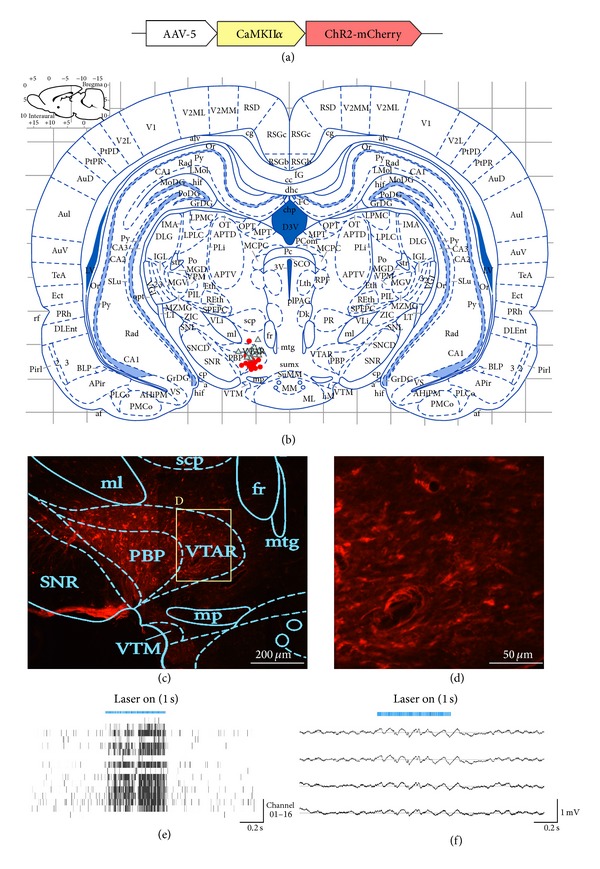
Histology and* in vivo* electrophysiology for verification of the optogenetic studies. (a) Sketch of the AAV vector used for the optogenetic transductions in this study. (b) The centers of viral-delivery regions (the red circular dots) and the placements of optical fiber tips (the blue triangular dots) for all the optogenetic rats (*n* = 6 for the free-moving tasks, *n* = 6 for the lever-pressing tasks, and *n* = 2 for erroneous displacements). These two series of dots were measured by individually locating the traces of optrode fiber tips and of the microinjector observed under microscopy. Both series of traces were measured in the targeted brain slices with AP = −4.8 mm from Bregma and overlapped into the atlas of Paxinos and Watson. (c) A typical view of the ChR2-mCherry expressions on the neurons expressing CaMKII*α* in and around the brain region of VTA that was overlapped with the brain atlas. (d) An inner set of (c) obtained from the region of interest (ROI) from the VTA region (including VTAR and PBP). ((e)-(f))* In vivo* electrophysiology recorded from the implanted optrode device. The blue bar shown in both figures represents a shot of laser stimulations with 15 ms pulse width, 50 Hz frequency, 1.0 s duration, and a light power around 1 mW. (e) presents spike activities from all the sixteen channels of the optrode device where each short straight bar represents one spike firing, and (f) is the LFP signals recorded from four typical channels of the optrode device.

The surgeries for viral delivery and optical fiber implantation were implemented via the optrode array device as described above. The rats were anesthetized with 1.0% sodium pentobarbital and again stereotaxically fixed. Adenoassociated viral vector serotype 5 (AAV-5) carrying the opsin gene of ChR2 and the gene for red fluorescent protein (mCherry) under CaMKII type-*α* promoter (Figure 1(a), AAV-CaMKII*α*-ChR2-mCherry, ~5 × 10^12^ titer, Neuron Biotech, Shanghai, China) was delivered into the rat brain using the microinjector (World Precision Instruments, Co., Ltd.) fixed on the stereotaxic apparatus. For each rat, 1.0 *μ*L of viral vector was injected through the guide cannula of the optrode array device into the left VTA (DV = −8.5 mm). The timing procedure of the viral injection was consistent with [11]. The microinjector and surgical apparatus were thoroughly sterilized after viral injection.

Upon finishing the viral delivery, an optical fiber with one-end FC tail was stereotaxically implanted through the guide cannula such that the fiber tip reached the dorsal edge of the VTA region (DV = −8.0 mm, 0.5 mm upper than the viral injection site) and the FC tail lay above the optrode cannula. Finally the optical fiber was covered up and mounted firmly on the entire array device using dental acrylic. The rats were kept for recovery and ChR2 expression for four weeks.

### 2.3. *In Vivo* Optical Stimulation and Electrophysiology

The optical instruments consisted of a 500 mW laser emitting 473 nm blue light (BL473T5-320FC, Shanghai Laser & Optics Century Inc., China) and a 3-meter optical fiber jumper with 50/125 multimodal glass optical fibers inside. The fiber jumper was connected to the laser and coupled to the optical fiber on the rat head by a plastic, tube-shaped FC-FC interface adapter [25]. The laser was triggered by transistor-transistor logic (TTL) pulses generated from a PG4000A digital stimulator (Cygnus Technology Inc., USA). The light power was measured by an optical power meter (LTE-1A, Chinese Academy of Sciences, Beijing, China). Usually a light power of 1–3 mW at the end of optical fiber jumper was approved for the following* in vivo* studies.

For* in vivo* electrophysiology, the electrical signals were recorded from the free behaving ChR2 rats via the Omnetics connector [25] jointed on the optrode device over the rat head. The signals were processed by a Plexon Multichannel Acquisition Processor (1–40 kHz rate, Plexon Inc., Dallas, TX). Signal preprocessing, including amplifications on programmable gain and filtering (set at 150 Hz–8 kHz band pass) for spikes, was implemented using the OmniPlex Controller as a part of the Plexon platform. Local field potentials (LFPs) were also recorded with band pass filtering at 10–170 Hz and digitized at 1 kHz sampling rate.

### 2.4. Design of Behavioral Experiments

#### 2.4.1. Free-Roaming Tasks with Field Tracking

The optogenetic rats were taken a series of behavioral experiments for free roaming in a circular field with 1.2 meters in diameter and were video-tracked throughout the experiments. The free-roaming tasks were given for 6 consecutive days. During each day, the rats were given three individual sessions, denoted as the* nonstim*,* optic stim*, and* optic blocking* sessions. For each session, the rats were allowed for 15 minutes free behaving to adapt the environment in the field with the optical fiber connected on its head, followed with a 10-minute test session: for the* nonstim* session, no laser stimulus was given; for the* optic stim* session, the rat was given a series of laser stimuli once every 0.6 second during the entire 10 min session with 15 ms pulse width, 50 Hz frequency, 0.2 s duration, and a light power at 1–3 mW; yet for the* optic blocking* session, the same laser stimuli were given, but the laser was blocked on its path at the FC interface over the rat head (using a thin ceramic chip). The rat movements were video-recorded across the whole test session for further analyses.

The video recorded from each session was processed for rat figure recognition using the OpenCV techniques [26] such that the rat locations during the test session were captured as the planar coordinate data (*x*, *y*) for the rat's gravity center. The data were then traced and jointed together to form a tracking map for free roaming in the field throughout the session (by MATLAB version 6.5, Mathwork software, USA), and the total intrasession distance of the free roaming rat was then calculated.

#### 2.4.2. Lever-Pressing Tasks

The lever-pressing tasks involved two groups of rats: the optogenetic rats (*n* = 6) with ChR2 expression in the VTA region and the control rats (*n* = 6) with microinjection of saline instead of the viral vector. The lever-pressing tasks were conducted for 6 consecutive days with a 30-minute test session per day for each rat. During the test session, the rat again connected with the optical fiber on its head was gently placed into the chamber for lever pressing with a fixed-ratio 1 (FR1) schedule: upon one lever press, the lever would automatically trigger the PG4000A stimulator and the laser device to generate a 1.0-second laser stimulus with the same pattern as in the free-roaming tasks. A video camera was mounted on the top for video recording. Basically, the rat pressing the lever with its forepaws was judged as a “correct” or an “active” press; the lever pressing or hitting by other parts of the rat body was regarded as an “incorrect” or “passive” press. Both the numbers of correct and incorrect lever presses were individually counted.

### 2.5. Histological Studies

The rats after the behavioral experiments were anesthetized with a lethal dose (240 mg/kg) of sodium pentobarbital and perfused transcardially with 300 mL saline at room temperature (RT) followed by 300 mL 4% paraformaldehyde (PFA). The head was removed following perfusion and embedded in 4% formaldehyde solution for two days at 4°C fridge. The brains were then transferred into 30% sucrose and embedded for one day before the frozen section. The brains were sectioned into 40 *μ*m coronal slices. The frozen brain slices were used for verification of the recording and optical stimulating sites by observing the different optrode traces inside the slices under a microscope. Fluorescent images were taken to identify the distribution of ChR2-mCherry expression in and around the left VTA region. Two sets of traces on the brain slices, one was the centers of viral delivery and the other was the tips of optrode fibers, were individually measured with the assistance of the atlas of Paxinos and Watson for neuroanatomical studies.

Immunohistochemistry was conducted with the 40 *μ*m coronal brain slices prepared in the same manner. The brain sections were rehydrated by free-floating in the phosphate buffer solution (PBS) for 15 minutes. Tissues were next incubated in −20°C pure methanol for 10 minutes. Thereafter the brain slices were incubated with the primary antibody (Rat antityrosine hydroxylase, Ab6211, Abcam, Cambridge, MA, USA) at a concentration of 1 : 500 diluted with 0.1% Triton X-100 and 4% Bovine serum albumin (BSA) in 4°C fridge over two days. After incubation with the primary antibody, the brain slices were then washed with PBS for 10 min × 5 times. The secondary antibodies (Alexa Fluor 488, rabbit anti guinea pig, Invitrogen, Carlsbad, CA, USA) at a concentration of 1 : 1000 in PBS were employed and the tissues were incubated for 2 hours at RT. Then the washing steps for the primary antibodies were repeated. The brain tissues were then carefully mounted on glass slides for later observations with confocal microscopy. For each optogenetic rat, part of the sections containing optrode traces was selected for detail analyses.

### 2.6. Data Analysis

Data acquired from the free-roaming studies were analyzed with two-way repeated measures ANOVAs, while Student's *t*-test was employed to compare the average distances across 6 sessions under the* nonstim*, the* optic blocking*, and the* optic stim* as well as the trained* optic stim* patterns individually, where *P* < 0.05 indicated statistical differences.

For the lever-pressing tasks, numbers of lever pressing during one 30-minute session for both optogenetic rats and control rats were individually averaged for each day and were mapped to time-course curves. The data of both groups for the six days were individually compared again using *t*-test with *P* < 0.05 indicating statistical differences.

## 3. Results

### 3.1. Optogenetic Expression and Optical Activation of Neural Activities in the VTA Region

For optogenetic transductions, the AAV-type-5 viral vectors carrying CaMKII*α*-ChR2-mCherry (see Figure 1(a)) were injected into the VTA region of the rat brains. Figures 1(c) and 1(d) provided typical views of the histological data, which revealed a high density of ChR2-mCherry expressions in the targeted VTA region (VTAR and PBP) with normal cell morphologies. The center of the opsin expressions (red dots in Figure 1(b)) and the location of the optical fiber end (blue dots in Figure 1(b)) of each rat were presented on the brain slices as shown in Figure 1(b). The assembles of trace labels revealed that the ChR2 optogenetic expressions were precisely located in the VTA region with the optrode fiber tip ~0.5 mm above for most of the rats, appropriate for light penetrating on the brain regions with opsin expressions.

The neuronal activities of the VTA neurons during optical stimulations were electrically recorded* in vivo* via the implanted optrode array devices. Figures 1(e) and 1(f) showed the spike firing activities and local field potentials recorded from multiple channels in the VTA region, respectively. The laser bars displayed in both Figures 1(e) and 1(f) indicated 473 nm laser delivery with the same stimulating pattern as in the free-roaming tasks (see Section 2.4.1). It was observed that a 1.0-second laser stimulus induced a significant increase in spike firing activities reflected in most channels (13 out of 16) from the array device, and after the end of laser stimulus the spike activity fell to the basic level. Similar results were observed in LFP changes where the field potentials exhibited a higher magnitude during the 1.0 s laser stimulus. Both results indicated that the* in vivo* optical stimulations on the optogenetic neurons enabled an increased neuronal activity in the VTA region.

### 3.2. The Optogenetic Rats Exhibited an Increased Locomotor Activity upon Light Stimulation during Free-Roaming Tasks

**Figure 2:**
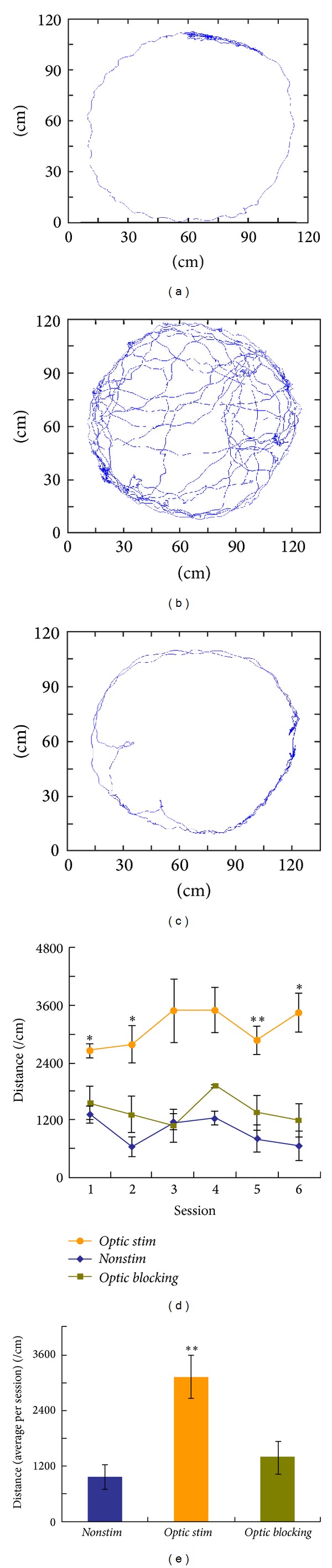
Data of the free-roaming tasks between the* nonstim*,* optic stim,* and* optic blocking* sessions. ((a)–(c)) Field tracking maps typically for the* nonstim*, the* optic stim*, and the* optic blocking* session, respectively. (d) Time courses of the intrasession distances for the above three sessions (see the figure legends). The error bar in each session dot represents the standard deviation of all the rats for the session (*n* = 6 for each session dot). Single asteroids represent statistical differences between the session dots of* optic stim* and* nonstim*, whereas double asteroids represent significant differences between them. (e) The results from the time course (d) were averaged and then bar-plotted over the three sessions. Double asteroids represent a significant difference compared to the results of* nonstim* sessions.

The optogenetic rats were performed with free-roaming behavioral tasks to illustrate the influence of optical stimulation on their locomotor activities. Two typical tracking maps of the rat in-field roaming during a* nonstim* and an* optic stim* session, respectively, were shown in Figures 2(a) and 2(b). Normally in the* nonstim *session the rat would exhibit a short-term roaming for exploration and then stay still against the edge wall for the rest of the session. While, in the* optic stim* session, the rat exhibited a more intense and long-term free roaming that it would travel more laps along the circular field. Also it was observed that during the* optic stim* session the rats took far more approaches into the center region of the field which is considered as an open field where normal rats seldom stay [27]. The time courses of the intrasession distances revealed statistical differences between* optic stim* and* nonstim* sessions for most days of free-roaming tasks (see Figure 2(d)). The bar plot of average intrasession distances across the six task days further indicated that the rats during* optic stim* sessions ran a significantly higher distance over* nonstim* sessions (see Figure 2(e)). The above data suggested that the rats under the optogenetic stimulation in VTA were able to exhibit a higher locomotor activity in the free-roaming tasks.

Although the rats had shown an increased locomotor activity in the above tasks, one might doubt that the “light leakage” during laser stimulations would influence on the behaving rats by light flashings over the rat eyes. An alternative series of free-roaming sessions with the laser blocked at the FC interface over the rat head (denoted* optic blocking*) was designed to eliminate the “light leakage” influences.  Figure 2(c) displayed the tracking map from a typical* optic blocking* session. Compared with Figure 2(a) from the* nonstim* session, little change was observed either on traveling laps or on approaches into the center field. The time courses of* optic blocking* sessions versus* nonstim* sessions revealed no statistical differences for the task days (see Figure 2(d)), and nor did the average bar plot show differences (see Figure 2(e)). Interestingly, it was observed from the plots that the* optic blocking* sessions held a slightly higher locomotion than the* nonstim* ones, yet with no statistical changes. This phenomenon was probably related to certain baseline properties of the rats' behavioral conditions, such as the curiosity to or being frightened with the frequent light flashings that we usually observed at the beginning of the free-roaming tasks. In general the data above suggested that simple light flashing over the rat eyes had no significant influences on the locomotor activity of the free behaving optogenetic rats. Totally, the behavioral data of free-roaming studies shown in Figure 2 supported our hypothesis that optogenetic activation of the excitatory neurons expressing CaMKII*α* in the VTA has positive reinforcement properties on the locomotor activity of free behaving rats.

### 3.3. Optogenetic Rats Achieved Better Performances in Lever-Pressing Tasks That Involves Reward Learning

**Figure 3:**
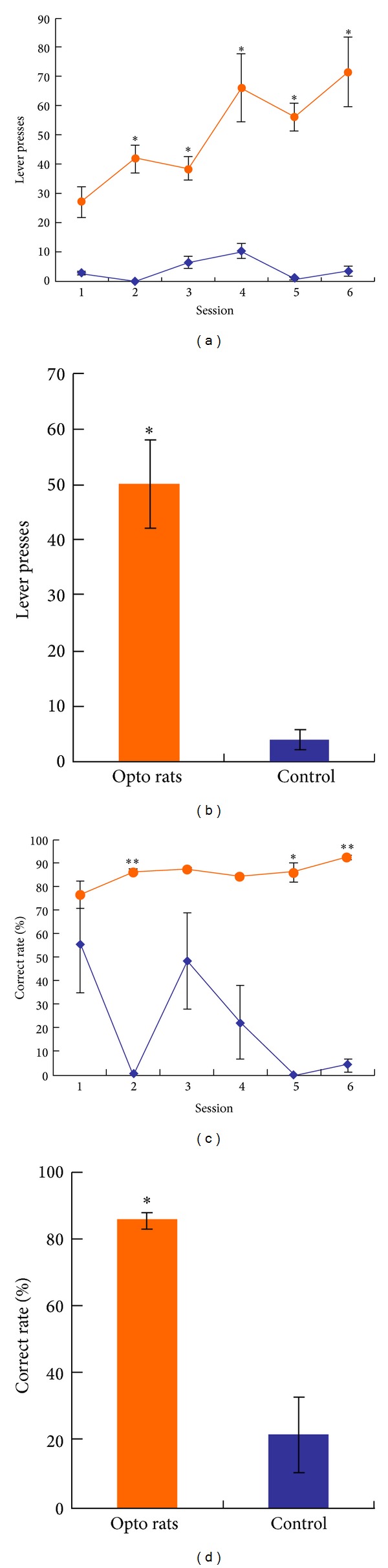
Data of the lever-pressing tasks for both optogenetic rats (denoted “opto rats”) and control rats. (a) Time courses of the total lever presses intrasession for opto rats and control rats individually (see the figure legends). (b) The total lever presses from (a) were averaged across all the six sessions and bar-plotted. (c) The correct rates, indicating the percentages of active lever presses within single sessions, were plotted as time courses for opto rats and control rats (see the figure legends). (d) Bar plot of the correct rates calculated from a total collection of lever-pressing data. For all the subfigures, the error bars in session dots or in bars represent the standard deviation of all the rats undertaking the session (*n* = 6 for each session dot or bar). Single asteroids represent statistical differences between the opto rats and controls, whereas double asteroids represent significant differences between them.

The underlying mechanisms of the optogenetic activation in the free-roaming tasks were probably depending on the reinforcing properties of VTA in the process of reward learning. Thereby we hypothesized that the optogenetic excitation of the VTA neurons expressing CaMKII*α* played a role of “virtual reward” that could induce reward seeking behaviors. For further investigation we conducted a set of lever-pressing tasks with optical self-stimulation in the VTA region for both optogenetic rats and controls as mentioned in Section 2.4.1. The data plotted in Figures 3(a) and 3(b) showed that the total intrasession lever presses of optogenetic rats were significantly higher than those of controls in most sessions. Also we note that the time course of optogenetic rats revealed an ascending tendency over the sessions. Besides, the plots of “correct rate” indicating the percentage of active lever presses during each session (see Figures 3(c) and 3(d)) showed that the optogenetic rats held a significantly higher correct rate than the controls for most sessions. Likewise, the time course of optogenetic rats in Figure 3(c) revealed an ascending tendency, whereas the course of controls appeared random with no tendency at all. In total, the data of lever-pressing tasks indicated that the optogenetic rats tend to make far more lever presses than the controls for obtaining the optical stimulation. Since the lever-pressing study is considered as a typical test for validation of reward learning, the results from the above data provided evidence to our hypothesis that the optogenetic activation of the excitatory neurons expressing CaMKII*α* in VTA played a role of virtual reward that led to a positive effect on the reward learning processes.

**Figure 4:**
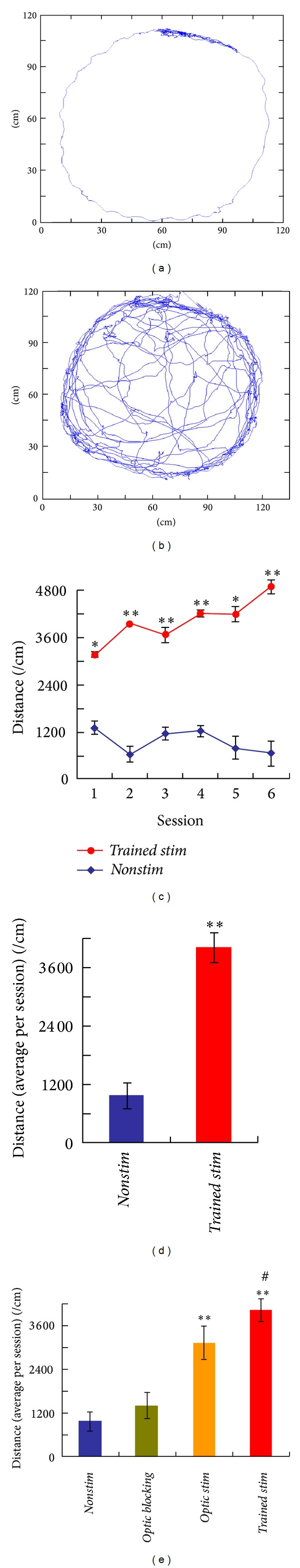
Data of free-roaming tasks combined with the* trained stim* sessions. (a and b) Field tracking maps of the* nonstim* and the* trained stim* session, respectively. Data and figures were acquired in the same manner as in Figure 2. (c) Time courses of the intrasession distances for both the* trained stim* and the* nonstim* sessions (see the figure legends). The error bar in each session dot represents the standard deviation of all the rats for the session (*n* = 6 for each session dot). Double asteroids represent significant differences between the session dots of* trained stim* and* nonstim*. (d) The intrasession distances were averaged from (c) and were bar-plotted over the* trained stim* and the* no-stim* sessions, where double asteroids represent a significant difference between them. (e) The bar plot of the average intrasession distances for all the four types of free-roaming sessions, the* nonstim*, the* optic blocking*, the* optic stim*, and the* trained stim*. Double asteroids represent significant differences compared to the* nonstim* data, while the sharp represents a statistical difference between the* optic stim* and the* trained stim* sessions.

Besides the learning of lever pressing, the learning of free roaming for obtaining the optical “reward” was also behaviorally conditioned on the optogenetic rats in this study. The procedures of training were basically the same with previous free-roaming tasks except that the laser stimulations were given manually once the rat started roaming. After six days of training, we repeated the free-roaming tasks on these trained rats over both* optic stim* (or the* trained stim*) and* nonstim* sessions. From the tracking maps (Figures 4(a) and 4(b)) a far more intense roaming in the* trained stim* session than the* nonstim* one appeared. Moreover, the time course of the* trained stim* sessions displayed a significant higher level over the* nonstim* one (see Figure 4(c)). Besides, a clear view of ascending tendency was observed from the* trained stim* course, which probably indicated the enhancement of reward learning processes of the rats. Both bar plots (Figures 4(d) and 4(e)) revealed that the average intrasession locomotion in the* trained stim* sessions was significantly higher than the* nonstim* ones and appeared the greatest among all the four types of sessions (*nonstim*,* optic blocking*,* optic stim*, and* trained stim*). Remarkably, the results of the* trained stim* sessions were statistically higher than those of the* optic stim* sessions before training, consistent with the properties of reward learning behaviors. Taken together, the above results suggested that reward learning took part in the mechanism underlying the optogenetic activation of the neurons expressing CaMKII*α* in the VTA of behaving rats and that by the reinforcement of reward learning, better performances on the rat locomotor activity were likely to be achieved.

### 3.4. The VTA Neurons under the Optogenetic Activation Were Partly Dopaminergic

**Figure 5:**
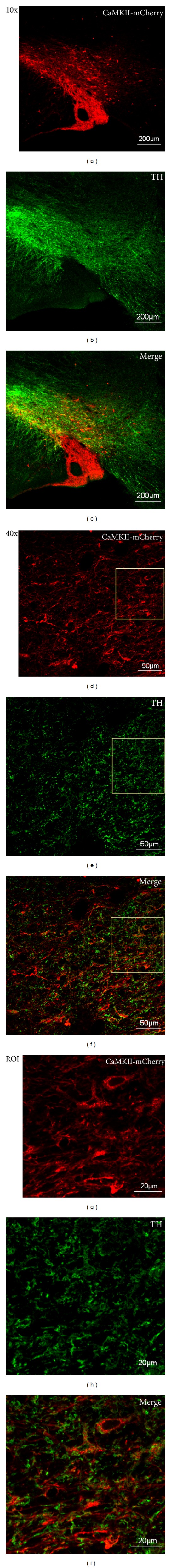
Immunochemistry results from the VTA region of brain slices from the optogenetic rats. All the subfigures were photographed under confocal microscopy and processed using the FV1000 software (Olympus Inc., Japan). The red channels represent CaMKII*α*-mCherry expressions and the green channels represent TH expressions, whereas the right column (Figures 5(c), 5(f), and 5(i)) indicates the merged two channels. ((a)–(c)) A typical view of the CaMKII*α*-mCherry and TH-GFP expressions under 10x microscopy. ((d)–(f)) A selected VTA region observed under 40x microscopy where the CaMKII*α* and TH expressions overlapped. ((g)–(i)) A region of interest (ROI) from the rectangular window shown in ((d)–(f)). The white arrows in (i) point out the several neuronal cell bodies merged with red and green channels, indicating that these neurons coexpress CaMKII*α* and TH.

In the above experiments we employed optogenetic manipulations on the excitatory neurons in the rat VTA region and conditioned a higher locomotor activity of free behaving rats based on the mechanism of reward learning. It is usually considered that dopaminergic (DA) neurons in the ventral striatum mediate in the reward learning mechanisms. Thus we investigated whether the DA neurons in VTA were selectively activated by the optogenetic manipulations in our study. The immunohistochemistry studies were employed to assess the locations of both the cell bodies labeling tyrosine hydroxylase- (TH-) GFP, a marker for DA neurons, and the cell bodies expressing CaMKII*α*-mCherry by optogenetic transduction in the VTA region.  Figure 5 displayed a typical set of the results. Figures 5(a)–5(c) revealed that both the expressions of CaMKII*α*-mCherry and TH-GFP were distributed in relative narrow area that contains the VTA. It was observed that the TH positive neurons appeared a higher density than the neurons expressing CaMKII*α*, consistent with previous reports that DA neurons held a predominant percentage in VTA [22]. Both the expressions of CaMKII*α*-mCherry and TH-GFP were ranged over the VTA region. Figures 5(d)–5(f) revealed that most TH positive neurons and CaMKII*α* positive neurons differed in cell morphologies. However, despite the fact that most TH positive neurons do not express CaMKII*α*, and many CaMKII*α* positive ones do not contain TH [19], there existed a substantial number of neurons expressing both CaMKII and TH (see Figure 5(f) and white arrows in Figure 5(i)). The immunohistochemistry data above indicated that part of the VTA neurons expressing CaMKII*α* corelease TH, thus exhibiting a dopaminergic property. These “dopaminergic” neurons appeared to take only a small percentage of the vast DA neurons within the VTA region.

## 4. Discussion

In this study we employed* in vivo* optogenetic transduction on the excitatory neurons in the brain region of VTA for adult rats by viral delivery of the AAV vector carrying ChR2 opsin genes. Our findings on the free-roaming tasks demonstrated that the optogenetic rats with ChR2 expression in the excitatory neurons expressing CaMKII*α* in VTA behaved at an increased locomotor activity upon 473 nm light activation while freely roaming in the circular field. This phenomenon was not likely owing to simple “light leak” effects with the blue light emitting over the rat eyes.

Further results on the lever-pressing tasks revealed that reward learning was mediating in the mechanisms underlying the CaMKII optogenetic activation in VTA in the above studies. These findings were consistent with previous reports that the activation of VTA neurons was able to induce reward learning behaviors for receiving the intracranial self-stimulations (ICSSs) [28]. Remarkably, in previous studies the highly frequent lever-pressing behaviors were typically induced by electrical stimulations, during which all the neural populations within the VTA were activated compulsively, making it unclear whether any type of subpopulations was involved in the reward learning behaviors. In this paper, however, we specifically activated the excitatory neurons expressing CaMKII*α* in the VTA by optogenetic manipulations and induced similar lever-pressing behaviors of the rats. These results indicated that these excitatory neurons expressing CaMKII*α* were probably a key population in the VTA that played a vital role in the conditioning of reward learning as in the lever-pressing tasks. Moreover, we repeated the free-roaming tasks on the optogenetic rats after a short-term training where light stimuli were given, while the rat was roaming forward, and observed that the rats performed a higher locomotor activity than before. These results further indicated that the reinforcement of reward learning placed a positive effect on the results of free-roaming tasks.

The immunohistochemistry further explored the nature of the excitatory neurons expressing CaMKII*α* in VTA under the optogenetic manipulation. The results revealed that those neurons expressing CaMKII*α* were partly collocated with dopaminergic neurons at the VTA region. The “collocated neurons” took only a small percentage of the VTA DA neurons, but they were observed in an appropriate area where light stimulations were reachable, which suggested that these neurons coexpressing CaMKII*α* and DA were activated upon light stimulations and probably took an active part in the behavioral conditionings in this study. Since the expressions of CaMKII*α* were widely implicated in glutamatergic neurons [1], those “collocated neurons” were likely to be neurons that corelease DA and glutamine in nature, consistent with previous reports that there exist a substantial area of excitatory neurons in the VTA region that corelease dopamine and glutamine and contribute to the functioning of reward learning and related behavioral conditionings [29, 30].

However, it was suggested from the immunohistochemistry results that there existed an alternative type of neurons that expresses CaMKII*α* but does not express TH. These neurons differed in cell morphology from the dopaminergic neurons and exhibited an expression of CaMKII*α*-mCherry. Based on the results above, it is inferred that those nondopaminergic neurons were probably simple glutamatergic neurons, consistent with previous reports that glutamatergic neurons were present in the VTA [19, 31]. According to previous reports, the exists a type of glutamatergic neurons in the VTA region that release VGluT2 but do not release dopamine, and they exhibit different electrophysiological properties and neuronal projections apart from the VTA neurons coexpressing glutamine and dopamine [32]. Moreover, these nondopaminergic neurons expressing CaMKII*α* were also located in VTA within the region of light activation, which indicated that these neurons were likely to take part in the optogenetic activations as well. Combined together, the excitatory neurons expressing CaMKII*α* in the VTA under optogenetic activations were composed of different types of neuronal subpopulation: one is the dopaminergic neurons that may corelease glutamine, and the other neurons were probably purely glutamatergic. Both types of neurons were likely to play vital roles in the behavioral conditionings as we conducted in this paper.

In conclusion, we employed optogenetic manipulations for free behaving animals based on viral transduction of ChR2 in the brain region of VTA and behavioral conditionings on reward learning. The results demonstrated that optogenetic activations of the excitatory neurons expressing CaMKII*α* in the VTA were able to upregulate the locomotor activity of free behaving rats and that by reinforcement of the reward learning behaviors the rats' locomotor activities were getting even enhanced. Further immunohistochemistry results revealed the nature of these VTA neurons expressing CaMKII*α*, which were partly dopaminergic that may play a vital role in the above behavioral conditionings. Future work may include further investigations on the nature of these neurons under the optogenetic activation in this study, such as the relationships between the neurons expressing CaMKII*α* and glutamatergic neurons in the VTA. Moreover, this study has provided a novel method to upregulate the locomotor activity by inducing the reward seeking behavior and by reward learning the “reward-motion” connectivity had gotten enhanced for the optogenetic rats, thus indicating further applications in the field of neural engineering such as animal robotics for precise control of animal behaviors.
